# Interleukin-6 mediated inflammasome activation promotes oral squamous cell carcinoma progression via JAK2/STAT3/Sox4/NLRP3 signaling pathway

**DOI:** 10.1186/s13046-022-02376-4

**Published:** 2022-05-05

**Authors:** Li Xiao, Xue Li, Peilin Cao, Wei Fei, Hao Zhou, Na Tang, Yi Liu

**Affiliations:** Department of Stomatology, Sichuan Provincial People’s Hospital, University of Electronic Science and Technology of China, Chengdu, 610072 Sichuan China

**Keywords:** IL-6, Oral squamous cell carcinoma, NLRP3 inflammasome, JAK2, STAT3, Sox4, NLRP3 pathway

## Abstract

**Background:**

Interleukin-6 (IL-6) has been reported to be critical in oral squamous cell carcinoma (OSCC). However, the set of pathways that IL-6 might activate in OSCC are not fully understood.

**Methods:**

IL-6 and Sox4 expressions were first determined with RT-qPCR, ELISA, Western blot, or immunohistochemistry in OSCC tissues, and correlations between IL-6 and Sox4 expression and patient pathological characteristics were examined, and Kaplan–Meier approach was employed for evaluating the prognostic utility in OSCC patients. CCK-8, EdU stain and colony formation assays were utilized to test cell proliferation in vitro. Mechanistically, downstream regulatory proteins of IL-6 were verified through chromatin immunoprecipitation, luciferase reporter, pull-down, and the rescued experiments. Western blot was used for detecting protein expression. A nude mouse tumorigenicity assay was used to confirm the role of IL-6 and Sox4 in vivo.

**Results:**

IL-6 was upregulated in OSCC tissues, and Sox4 expression was positively correlated with IL-6 expression. High IL-6 and Sox4 expression was closely related to tumor size, TNM stage, and a poorer overall survival. Besides, IL-6 could accelerate OSCC cell proliferation by activating inflammasome via JAK2/STAT3/Sox4/NLRP3 pathways in vitro and in vivo. Furthermore, STAT3 played as a transcription factor which positively regulated Sox4, and IL-6 promotes Sox4 expression by activating JAK2/STAT3 pathway. Moreover, through the rescue experiments, we further confirmed that IL-6 could promote proliferation and NLRP3 inflammasome activation via JAK2/STAT3/Sox4 pathway in OSCC cells. Finally, knockdown of Sox4 suppressed OSCC growth in vivo, and antagonized the acceleration of IL-6 on tumor growth.

**Conclusions:**

We confirmed that IL-6 plays an oncogenic role in OSCC progression by activating JAK2/STAT3/Sox4/NLRP3 pathway, which might be the therapeutic targets for OSCC remedy.

**Supplementary Information:**

The online version contains supplementary material available at 10.1186/s13046-022-02376-4.

## Background

Oral squamous cell carcinoma (OSCC) is a familiar malignant tumor, accounting for about 95% [1]. There are more than 300,000 new cases of OSCC yearly, and the age of onset displays a decreasing trend year by year [2]. Currently, the pathogenesis of OSCC remains unclear. Bad habits, such as smoking and drinking alcohol, are the risk factors for OSCC, and other risk factors also contain HPV infection, chewing areca, and immune deficiency [3]. At present, the therapy of OSCC is mainly radical surgical resection, combined with comprehensive remedy [4]. Although the therapy for OSCC has improved, there has been no significant reduction in mortality, and the 5-year survival rate is only 50%-60%. OSCC, as an aggressive epithelial tumor, is associated with aberrant expression of multiple genes. Therefore, it is particularly critical to verify the mechanism of OSCC to improve OSCC remedy.

Interleukin-6 (IL-6) can act on cell receptors through autocrine or paracrine pathways to induce intercellular signal transduction and exchange, thus completing the relevant biological functions of cells [5, 6]. There are two pathways of IL-6 transduction: classical pathway and trans-pathway. In the classical pathway, IL-6 can directly bind to IL-6R on cell membrane to mediate cell signal transduction [6, 7]. In the trans-pathway, IL-6 can bind to sIL-6R and interacts with gpl30 to activate the downstream pathways including Janus protein tyrosine kinase (JAK), STAT3, MAPK, P13K, etc. [8]. Researches testified that IL-6 is relevant to tumor growth, differentiation, apoptosis, drug resistance, and immune regulation, etc. IL-6 was also reported to be notably elevated in patients with advanced tumors [9, 10]. And IL-6, as a diagnostic and therapeutic target, plays crucial roles in the OSCC progression, such as radio resistance, proliferation, and metastasis, etc. [11–14]. JAK/STAT pathway is one of the most crucial cytokine signal transduction pathways and extensively involved in the regulation of multiple pathophysiological processes, especially inflammatory responses [15, 16]. Activation of STAT3 can bind to target genes to change gene expression, and further participate in cell renewal, survival, division, metastasis, and other processes [17, 18]. Study certified that JAK/STAT pathway could participate in the migration and apoptosis of OSCC cells [19]. Besides, IL-6/JAK/STAT3 axis has also been discovered to have pleiotropic effects in cancers, including gastric cancer [20], colorectal cancer [21], and breast cancer [22], etc. However, it is not clear whether IL-6 can influence OSCC progression through JAK/STAT3 pathway. And the detailed downstream regulatory mechanism of IL-6/JAK/STAT3 axis is not elucidated in OSCC.

In this research, we first confirmed the expressions of IL-6, IL-1β, and IL-18 in OSCC tissues. Meanwhile, we also identified the impacts of IL-6 on proliferation capacity in OSCC cells and xenograft tumor of model mice. Besides, we verified that whether JAK/STAT3 is a key regulatory pathway in OSCC proliferation mediated by IL-6. Moreover, we further investigated the possible mechanisms by which the IL-6/JAK/STAT3/Sox4 axis could affect OSCC progression. Therefore, our study preliminarily revealed the importance of IL-6/JAK/STAT3/Sox4 axis in the progression of OSCC and its potential regulatory mechanism, which might be effective approaches for the therapy of OSCC.

## Materials and methods

### Patients and samples

Twenty normal oral mucosa tissues, forty oral lichen planus (OLP) tissues and 108 cases of OSCC tissues were harvested from patients or volunteers in the Sichuan Provincial People’s Hospital from 2010 to 2015 and stored at -80 °C in the refrigerator for further analysis. Among the 108 patients, 9 patients were progressed from OLP to OSCC. At the same time, we collected peripheral venous blood (5 mL) from these participants, and the supernatant was collected by centrifugation (4 °C, 3 000 × g/min, 20 min). The supernatant was divided into sterile EP tubes and stored at -80 °C in the refrigerator. Inclusion criteria: The diagnosis of OSCC was confirmed by pathological sections and clinical diagnosis; all were first-time cancer patients with complete pathological data; no radiotherapy, chemotherapy and drug treatment; patients and their relatives agreed and signed the informed consent. Exclusion criteria: combination of tumors from other sites; combination of hypertension, diabetes mellitus; patients during pregnancy or lactation. In addition, clinical findings and follow-up data pertaining to these patients were recorded. The study was approved by Ethics Committee of Sichuan Provincial People’s Hospital [Sichuan, P.R. China; approval no. 2015NSF(7)], all patients signed informed consent.

### Cell culture

Oral epithelial cells (HOEC) and OSCC cell line Cal27 were purchase from Procell (Wuhan, China), OSCC cell lines (SCC-4, SCC-15, SCC-9, SCC-25, and Tca83) were from ATCC (Manassas, VA). All cells were hatched in DMEM (Gibco) with L-glutamine, sodium pyruvate, 10% fetal bovine serum (FBS, Sigma) at 37 °C and 5% CO_2_.

### Cell treatment and transfection

SCC-15 and SCC-25 cells were first addressed with 0, 0.1, 1, 5, 10, 25, 50 ng/mL IL-6 for 24 h, respectively. SCC-15 and SCC-25 cells were also processed with 25 ng/ml IL-6, 5 μmol/L JAK2 inhibitor (Fedratinib) [23], and 25 μmol/L STAT3 inhibitor (Protosappanin A) for 24 h [24]. Empty vector (pcDNA4.0), Sox4 overexpression plasmid (pcDNA4.0-Sox4), Sox4 shRNAs (shSox4, 5’-GCGACAAGATCCCTTTCATTC-3’), NLRP3 overexpression plasmid (pcDNA4.0-NLRP3), NLRP3 shRNAs (shNLRP3, 5’-GCTTCATCCACATGACTTTCC-3’), and negative control (NC) shRNAs were gained from HanBio Biotechnology (HanBio, Shanghai, China). SCC-15 and SCC-25 cells (1 × 10^5^ cells/well) in 6-well plates were transfected with shSox4, pcDNA4.0-Sox4, shNLRP3, or pcDNA4.0-NLRP3 for 48 h using lipofectamine 3000 (Invitrogen) in accordance with the specification.

### RT-qPCR

The processed OSCC cells were harvested or the tumors in each group were ground, and total RNAs were isolated applying TRIzol reagent (Invitrogen, MA, USA). Subsequently, cDNAs were synthesized with the RNAs (as template) and PrimeScript™ RT reagent Kit (TaKaRa). Then PCR amplification was then conducted with SYBR Green qPCR Master Mix (DBI Bioscience) after reverse transcription. The data in this experiment were calculated with 2^−△△CT^ method, and GAPDH was the internal control. The primers for different genes are given as followed: IL-6: 5’-ACTCACCTCTTCAGAACGAATTG-3’ (forward) and 5’-CCATCTTTGGAAGGTTCAGGTTG-3’ (reverse); IL-6R: 5’-CCCCTCAGCAATGTTGTTTGT-3’ (forward) and 5’-CTCCGGGACTGCTAACTGG-3’ (reverse); Sox4: 5’-AGCGACAAGATCCCTTTCATTC-3’ (forward) and 5’-CGTTGCCGGACTTCACCTT-3’ (reverse); NLRP3: 5’-GATCTTCGCTGCGATCAACAG-3’ (forward) and 5’-CGTGCATTATCTGAACCCCAC-3’ (reverse); GAPDH: 5’-CTGGGCTACACTGAGCACC-3’ (forward) and 5’-AAGTGGTCGTTGAGGGCAATG-3’ (reverse).

### Western blot

The processed OSCC cells and the ground tumors were increased with the lysates including RIPA (Beyotime, China) and protease inhibitors (Beyotime, China). The extracted proteins were quantified through BCA method, mixed with appropriate loading, and heated at 100℃ for denaturation. Then same amount (50 μg) of proteins were added to 10% SDS-PAGE, separated by electrophoresis at constant pressure, and transferred to PVDF membrane (Millipore). Next, the membrane with protein was sealed with 5% skim milk for 2 h, exposed to primary antibodies, including ASC (Abcam, ab283684, 1:1000), IL-1β (Abcam, ab254360, 1:1000), IL-18 (Abcam, ab207324, 1:1000), Pro-IL-18 (Proteintech, 10,663–1-AP, 1:1000), NLRP3 (Abcam, ab263899, 1:1000), IL-6 (Abcam, ab9324, 1 µg/ml), Sox4 (Abcam, ab70598, 1:500), JAK2 (Abcam, ab108596, 1:1000), pJAK2 (Abcam, ab32101, 1:1000), STAT3 (Abcam, ab68153, 1:1000), pSTAT3 (Abcam, ab267373, 1:1000) at 4℃ overnight, and secondary antibodies (Abcam) for 1 h. Finally, western blotting was developed after processing with ECL kit (Thermo scientific), and the brightness of each strip can be controlled by adjusting the exposure time.

### ELISA assay

In line with the instruction, IL-1β ELISA kit (Abcam, ab214025 [for human] and ab197742 [for mouse]) and IL-18 ELISA kit (Abcam, ab215539 [for human] and ab216165 [for mouse]) was utilized to test IL-1β and IL-18 activities.

### CCK-8

The treated OSCC cells were evenly increased into 96-well plates (100 μL, 1 × 10^3^ cells/well). Then cells were growth in a 37 ℃ incubator and each well was supplemented with 10 μL CCK-8 (Dojindo, Tokyo, Japan) at 0, 24, 48, and 72 h. After additional 2 h of incubation, the OD was monitored by applying a microplate reader (Bio-TekEpoch) at 450 nm.

### Colony formation assay

The processed OSCC cells (1 × 10^3^ cells) were routinely hatched in a 6-well plate at 37 ℃ for 14 days. Then the adherent clones were fixed and dyed using 0.1% crystal violet, and clones were counted.

### EdU staining

The processed OSCC cells (1 × 10^5^ cells/well) were incubated in 6-well plates until they adhere to the wall. Then cells were processed with 100 μL EdU regent (50 μM, Life Technologies) for 2 h at 37 °C. Then cells were fixed using 4% formaldehyde (30 min), disposed of 50 μL 2 mg/mL glycine and 100 μL 0.5 Triton X-100. The Edu-positive cells were photographed using a fluorescence microscope (Olympus, Tokyo, Japan).

### Chromatin immunoprecipitation (ChIP) assay

SCC-15 cells (1 × 10^6^ cells/dish) were uniformly placed in 10 cm cell culture dishes and fixed using 37% formaldehyde at 37 ℃ for 10 min. Then cells were disposed of 0.125 M glycine and SDS Lysis Buffers, and DNA was interrupted into 200–1000 bp through ultrasound. Next, the mixture was addressed with 8 μL NaCl (5 M) at 65 ℃ and cross-linked for 4 h. Subsequently, GenClean Agarose Gel DNA Recovery Kit was applied to extract DNA, and co-precipitation was conducted with the CHIP kit (Millipore). The Sox4 primers for ChIP as followed: forward 5’-GCACCAGAGGCTGATTCT-3’ and reverse 5’-CTGCTTAAAAGCCAAGTG-3’.

### Luciferase reporter assay

The downstream target genes for transcription factor STAT3 were identified by JASPAR 2022 (https://jaspar.genereg.net/). We first constructed the wild type (WT) and mutant (Mut) Sox4 promoter plasmids (pGL4.1-Sox4-promoter-WT or pGL4.1-Sox4-promoter-MUT) by referring to the binding sites between STAT3 and Sox4 promoter region. 293 T cells were then co-transfected with the corresponding plasmids and internal plasmids (pTK-RL) containing the Renilla luciferase gene by applying Lipofectamine 3000 (Invitrogen). After 48 h of transfection, luciferase (Firefly/Renilla) activity was measured using the Dual luciferase assay kit (Promega).

For NLRP3, the wild type promoter of NLRP3 was constructed (pGL4.1-NLRP3-promoter-WT). SCC-15 and SCC-25 cells were then co-transfected with pGL4.1-NLRP3-promoter-WT plasmids, different concentration of Sox4 overexpression plasmids (0, 0.1, 0.5, 1, 5, 10, 20, 50 ng) and internal plasmids (pTK-RL) containing the Renilla luciferase gene by applying Lipofectamine 3000 (Invitrogen). After 48 h of transfection, luciferase (Firefly/Renilla) activity was measured using the Dual luciferase assay kit (Promega).

### DNA Pull-down assay

The interaction between STAT3 and Sox4 promoter was confirmed by applying a DNA pull-down test kit (Gzscbio, Guangzhou, China). Briefly, cells were lysed after centrifugation. And then we mixed the streptavidin magnetic beads (125 μL) and probes targeting Sox4 promoter (25 μL, 8 μmol/L). And the mixture continued to mix with cell lysis for 12 h on ice. After elution, the conjunct protein was collected and Western blot was utilized to test STAT3 expression.

### Tumor xenograft model

BALB/c male nude mice (SPF, 4 weeks, 20 ± 2 g) were obtained from Shanghai slack laboratory. And the experimental mice were fed through separate cages under the conditions of humidity 45%-55%, temperature 22–25 ℃, light for 12 h, adequate standard feed, and purified water. After one week, SCC15 cells were harvested and counted, and the right anterior axilla of each mouse was subcutaneously injected with 2 × 10^5^ cells in 0.2 mL PBS. To evaluate the role of IL-6 on tumor growth, when the tumor volume reached 150 mm^3^, the tumor xenograft was directly injected with 100 μl PBS (Sigma-Aldrich, P2272, pH = 7.2), 10 mg/kg IL-6 recombinant protein (Fully biologically active, Abcam, ab259381) in 100 μl PBS (Sigma-Aldrich, P2272, pH = 7.2), or 10 mg/kg IL-6 antibody (Abcam, ab259341) in 100 μl PBS (Sigma-Aldrich, P2272, pH = 7.2) once a week, and the mice were divided into control, PBS, IL-6 group and IL-6 antibody groups. To evaluate the role of Sox4 on tumor growth, SCC15 cells were transfected with shSox4 and a Sox4 knockdown stable cell line was constructed. Each mouse was subcutaneously injected with 2 × 10^5^ SCC15 cells with Sox4 knockdown in 0.2 mL PBS. when the tumor volume reached 150 mm^3^, the tumor xenograft was directly injected with 10 mg/kg IL-6 recombinant protein (Fully biologically active, Abcam, ab259381) in 100 μl PBS (Sigma-Aldrich, P2272, pH = 7.2), and the mice were divided into control, shSox4, IL-6 group and shSox4 + IL-6 groups. Tumor length was tested every 7 days for 21 days. At 21 days, the BALB/c nude mice were dislocated and sacrificed, and the tumors were collected. All animal experiments were done in animal laboratory center as per the study protocol according to the NIH Guide for the Care and Use of Laboratory Animals, approved by the Animal Care and Use Committee of the Sichuan Provincial People’s Hospital.

### Immunohistochemistry (IHC)

Normal oral mucosa, OLP and OSCC tissues from human and the tumors from mice were fixed in 10% formaldehyde for 24 h. All tissues were conventionally dehydrated, paraffin embedded, and cut into 4 μm thick slices. Then the slices were conventionally dewaxed, washed, and repaired using sodium citrate (pH = 6.0) for 8 min under high temperature and high pressure. After washing, the slices were added into 3% H_2_O_2_ and then heated. Subsequently, the slices were blocked using 10% BSA, placed at 4℃ overnight with anti-IL-6, anti-Sox4, anti-NLRP3, anti-Ki-67, followed by secondary antibody (Abcam) for 1 h. After washing, the slices were then subjected to multiple processing in the later stage, including DAB treatment (10 s), washing, hematoxylin redyeing (30 s), dehydration, neutral gum sealing and natural drying. The results were obtained under a light microscope.

### Statistical analysis

All experiments were conducted in thrice, and all data was displayed with mean ± SD and counted with SPSS 21.0 (SPSS, Inc.). And the statistical charts were made with GraphPad Prism 8.0. And the paired Student's t test or One-Way ANOVA were utilized for statistical calculations. The curves of Kaplan–Meier and log-rank assessments were employed to evaluate differences in survival outcomes between groups, while associations between IL-6 and Sox4, IL-1β or IL-18 were appraised through Pearson correlation assessments. Chi-square test was adopted to determine the relation between IL-6 or Sox4 expression level and clinical characteristics of OSCC patients. *P* < 0.05 represented the statistical significance.

## Results

### Sox4, IL-1β, and IL-18 were upregulated and positively correlated with IL-6 in OSCC tissues

**Fig. 1:**
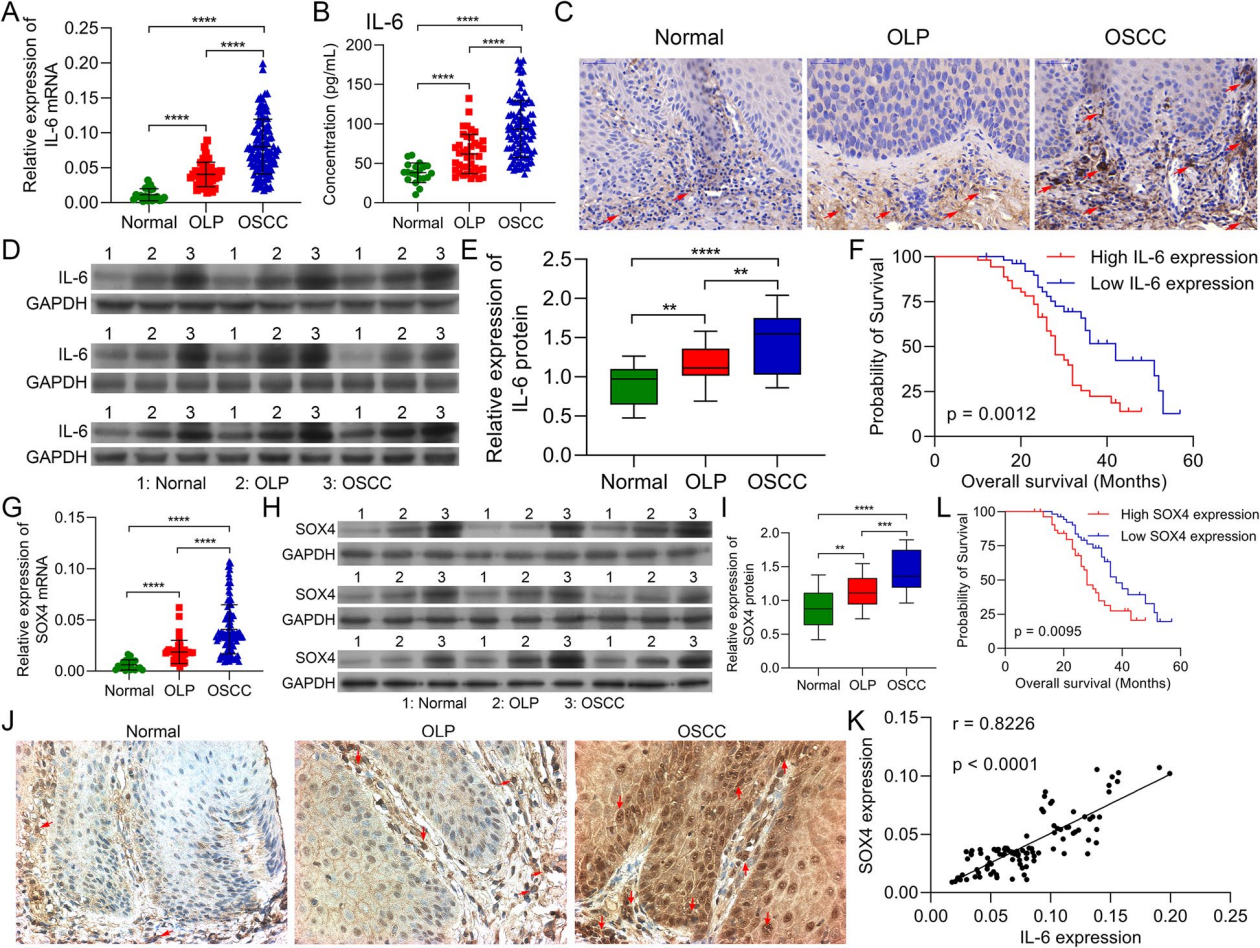
IL-6 and Sox4 were memorably up-regulated, and positively correlated in OSCC tissues. (**A**) RT-qPCR analysis of IL-6 in normal, OLP, and OSCC tissues. (**B**) The content of IL-6 was tested with ELISA kit in the serum of normal, OLP, and OSCC patients. (**C**) IL-6 expression was determined via IHC in normal, OLP, and OSCC tissues. (**D** and **E**) Western blotting analysis of IL-6 in normal, OLP, and OSCC (developed after follow-up) tissues, which were from the same patient (*n* = 9). After diagnosis of OLP, OLP tissue and adjacent normal tissue were taken, and OSCC tissue was taken after follow-up. (**F**) The overall survival of IL-6 expression in OSCC patients. (**H**) The level of Sox4 was confirmed via RT-qPCR. (**H** and **I**) Sox4 expression levels were assessed with Western blot in normal, OLP, and OSCC tissues, which also were from the same patient (*n* = 9). (**L**) The overall survival of Sox4 expression in OSCC patients. (**J**) Immunohistochemical staining of Sox4 expression in normal, OLP, and OSCC tissues. (**K**) Correlation analysis of IL-6 and Sox4 (*r* = 0.8226). ** *P* < 0.01, *** *P* < 0.001, **** *P* < 0.0001

To verify the expression changes and prognosis of IL-6 and Sox4 in OSCC patients, we first collected 20 cases of normal oral mucosa tissues, 40 cases of OLP tissues, and 108 cases of OSCC tissues. RT-qPCR data signified that IL-6 mRNA level was raised in OLP tissues versus that in normal oral mucosa tissues, and IL-6 was also upregulated in OSCC tissues versus that in normal and OLP tissues (Fig. 1A). And ELISA data also denoted that compared with normal and OLP patients, the concentration of IL-6 was also memorably heightened in the serum of OSCC patients (Fig. 1B). Similarly, IHC data manifested that IL-6 protein was signally aggrandized in OSCC tissues relative to that in normal and OLP tissues, and IL-6 level was lowest in normal oral mucosa tissues (Fig. 1C). And western blot also exhibited that the level of IL-6 was distinctly increased in OSCC tissues (Fig. 1D and 1E). Moreover, high expression of IL-6 was relevant to large tumor size (*P* = 0.0123) and TNM stage (*P* = 0.0002) in 108 cases of OSCC patients (Table 1). Besides, we discovered that high expression of IL-6 displayed a short survival time in OSCC patients (Fig. 1F). Thus, we concluded that IL-6 was upregulated and was related to the poor prognosis in OSCC patients.

**Table 1:** Correlation between IL-6 expression and clinicopathological features in 108 cases of OSCC

Factors	*N*	IL-6 expression (*n* = 108)		*P* value
		Low expression (*n* = 54)	High expression (*n* = 54)	
**Age**				
< 60	49	23	26	0.5620
≥ 60	59	31	28	
**Gender**				
Male	52	25	27	0.7001
Female	56	29	27	
**Tumor size (cm)**				
< 3	53	33	20	**0.0123**
≥ 3	55	21	34	
**TNM stage**				
I—II	45	32	13	**0.0002**
III—IV	63	22	41	
**Differentiation**				
Poor	48	20	28	0.1213
Well	60	34	26	

Meanwhile, our data represented that Sox4 level was also dramatically elevated in OSCC tissues versus that in normal oral mucosa and OLP tissues, and Sox4 expression in OLP tissues was higher than that in normal oral mucosa (Fig. 1G-J). Moreover, through correlation analysis, we discovered that IL-6 expression was positively related to and Sox4 in OSCC patients (*r* = 0.8226, Fig. 1K). Meanwhile, we discovered that high expression of Sox4 was relevant to large tumor size (*p* = 0.0039) and TNM stage (*p* = 0.0034) in 108 cases of OSCC patients (Table 2). And high expression of Sox4 also indicated a short survival time in OSCC patients (Fig. 1L). We concluded that Sox4 was upregulated in OSCC and relevant to IL-6 expression and OSCC poor prognosis.

**Table 2:** Correlation between Sox4 expression and clinicopathological features in 108 cases of OSCC

Factors	*N*	Sox4 expression (*n* = 108)		*P* value
		Low expression (*n* = 54)	High expression (*n* = 54)	
**Age**				
< 60	49	22	27	0.3338
≥ 60	59	32	27	
**Gender**				
Male	52	23	29	0.2479
Female	56	31	25	
**Tumor size (cm)**				
< 3	53	34	19	**0.0039**
≥ 3	55	20	35	
**TNM stage**				
I—II	45	30	15	**0.0034**
III—IV	63	24	39	
**Differentiation**				
Poor	48	22	26	0.4386
Well	60	32	28	

Furthermore, ELISA data represented that IL-1β level was also observably increased in OSCC patients relative to that in normal and OLP patients (Figure S1A). And IL-1β concentration was also positively correlated with IL-6 concentration in OSCC patients (Figure S1B). Likewise, our data denoted that IL-18 was prominently elevated in OSCC patients, which was also positively associated with IL-6 concentration (Figure S1C and D). So, these data revealed that IL-6, Sox4, IL-1β, and IL-18 were all upregulated in OSCC tissues, and IL-6 manifested a positive correlation with Sox4, IL-1β, and IL-18.

### Introduction of IL-6 dramatically induced proliferation, and activated JAK2/STAT3Sox4/NLRP3 pathway in OSCC cells

**Fig. 2:**
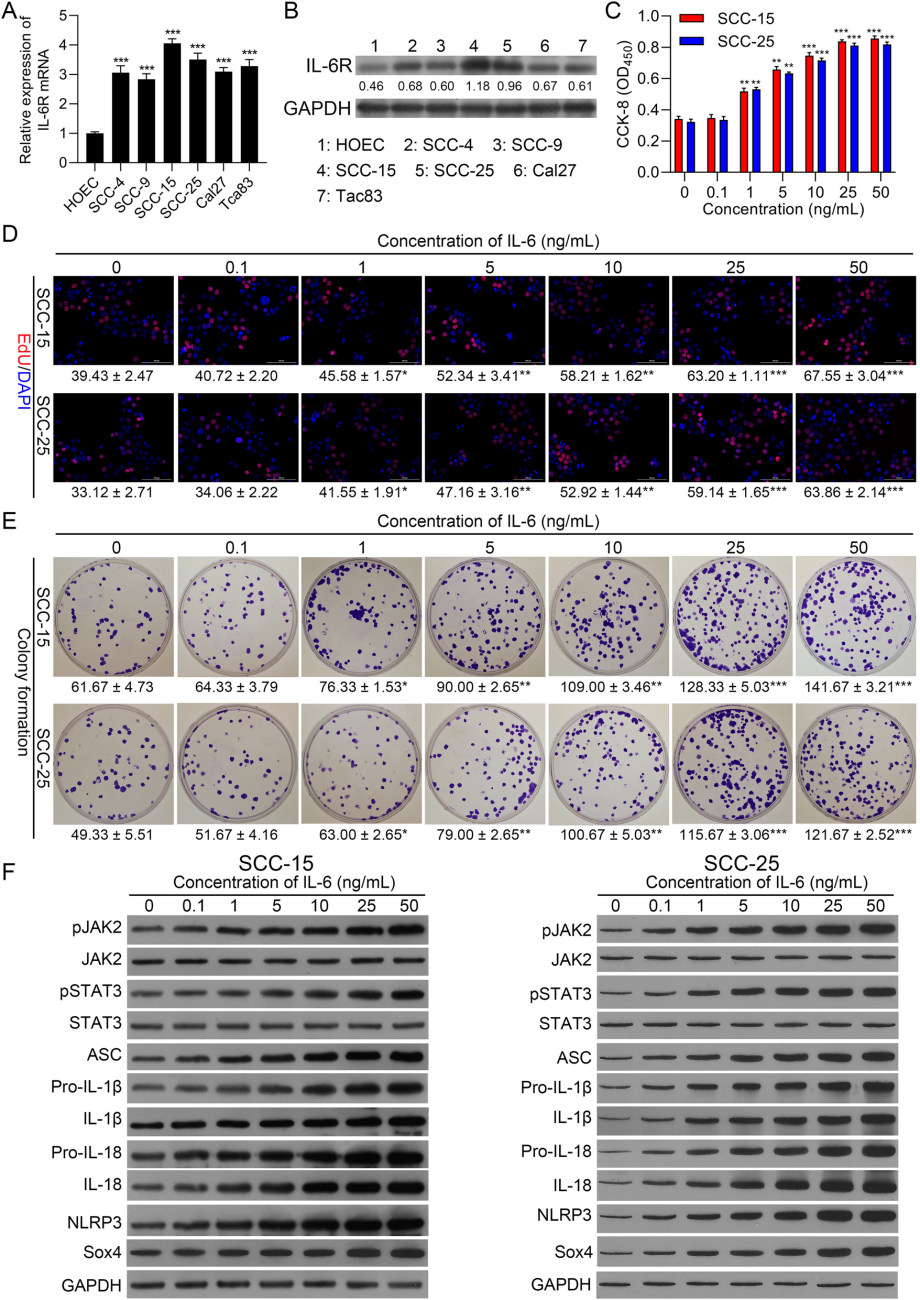
Introduction of IL-6 dramatically induced proliferation and activated JAK2/STAT3/Sox4/NLRP3 pathway in OSCC cells. IL-6R expression was credited through RT-qPCR (**A**) and Western blot (**B**) in oral epithelial cells (HOEC) and OSCC cell lines (SCC-4, SCC-9, SCC-15, SCC-25, Cal27 and Tca83). SCC-15 and SCC-25 cells were induced by 0, 0.1, 1, 5, 10, 25, 50 ng/mL IL-6, and cell proliferation was monitored by applying (**C**) CCK-8, (**D**) EdU staining and (**E**) colony formation. (**F**) Western blot was conducted to identify the impacts of IL-6 on pJAK2, JAK2, pSTAT3, STAT3, ASC, pro-IL-1β, IL-1β, pro-IL-18, IL-18, and NLRP3 in OSCC cells. ** *P* < 0.01, ***P < 0.001

To further determine the function of IL-6, and the possible protein pathways it might regulate in OSCC progression, we first identified IL-6 level receptor (IL-6R) in OSCC cells. As displayed in Fig. 2A, IL-6R was signally elevated in OSCC cells versus that in HOEC cells, especially, SCC-15 and SCC-25 cells. Similarly, western blot data also denoted that the expression trend of IL-6R protein was basically consistent with its mRNA (Fig. 2B). Based on these data, SCC-15 and SCC-25 cells were selected for future study. Subsequently, to further screen the appropriate concentration of IL-6 treatment, SCC-15 and SCC-25 cells were disposed of 0, 0.1, 1, 5, 10, 25, 50 ng/mL IL-6. CCK-8 data signified that cell viability was gradually aggrandized by IL-6 as its concentration rises in OSCC cells (Fig. 2C). Meanwhile, the data from EdU staining and colony formation also represented that the increase of IL-6 also could gradually enhance the proliferation in OSCC cells (Fig. 2D and E). As well, western blot results manifested that IL-6 treatment could observably heighten pJAK2, pSTAT3, Sox4, and NLRP3-related proteins (ASC, pro-IL-1β, IL-1β, pro-IL-18, IL-18, and NLRP3) expressions in OSCC cells, and the expressions of these proteins were also gradually increased with the elevation of IL-6 concentration (Fig. 2F). Besides, ELISA data also displayed that the concentrations of IL-1β and IL-18 were also could be raised by IL-6, especially 25 and 50 ng/mL IL-6 in OSCC cells (Figure S2). Overall, these results certified that IL-6 might facilitate proliferation and activated JAK2/STAT3/Sox4/NLRP3 pathway in OSCC cells. And based on the effects of different concentrations of IL-6 in SCC-15 and SCC-25 cells, we selected 25 ng/mL IL-6 in subsequent experiments.

### IL-6 observably accelerated proliferation and NLRP3 inflammasome activation by JAK2/STAT3 pathway in OSCC cells

**Fig. 3:**
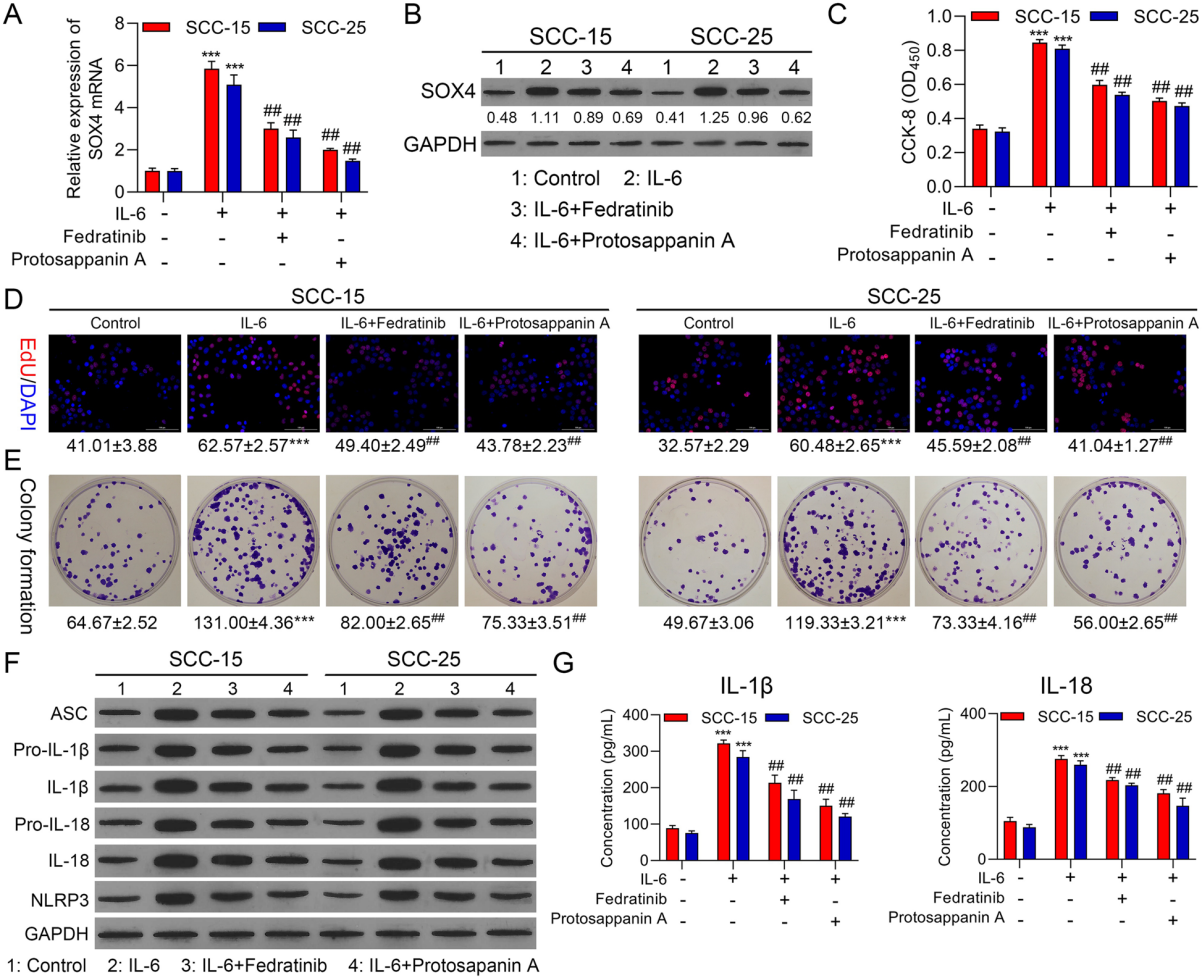
IL-6 observably accelerated proliferation and NLRP3 inflammasome activation by JAK2/STAT3 pathway in OSCC cells. SCC-15 and SCC-25 cells were processed with 5 μmol/L JAK2 inhibitor (Fedratinib) and 25 μmol/L STAT3 inhibitor (Protosappanin A), and induced by 25 ng/ml IL-6 for 24 h. The expression change of Sox4 was monitored using RT-qPCR (**A**) and Western blot (**B**). Cell proliferation was also tested using (**C**) CCK-8, (**D**) EdU staining and (**E**) colony formation. (**F**) The levels of inflammation-related proteins were assessed with Western blot. (**G**) ELISA kits were adopted to examine the concentration of IL-1β and IL-18 in the processed OSCC cells. *** P < 0.001 vs. control group; ## *P* < 0.01 vs. IL-6 group

In view of the above results, IL-6 could upregulate pJAK2 and pSTAT3. we further verified whether IL-6 could expedite cell proliferation and NLRP3 inflammasome activation through JAK2/STAT3 pathways. We also adopted JAK2 inhibitor (Fedratinib) and STAT3 inhibitor (Protosappanin A) to treat SCC-15 and SCC-25 cells, which were also processed with 25 ng/ml IL-6. We first discovered that Sox4 expression was markedly strengthened in IL-6 group relative to that in control group, while the upregulation of Sox4 also could be memorably weakened by Fedratinib or Protosappanin A treatment in OSCC cells (Fig. 3A and B). Besides, we testified that increase of IL-6 caused a remarkable enhancement in the proliferation of OSCC cells, which also could be notably attenuated by Fedratinib or Protosappanin A treatment (Fig. 3C-E). Moreover, we certified that IL-6 also could result in prominent upregulations in NLRP3-related proteins, which also could be dramatically subsided by Fedratinib or Protosappanin A treatment in OSCC cells (Fig. 3F). Likewise, ELISA results exhibited that Fedratinib or Protosappanin A treatment could prominently reduce IL-1β and IL-18 levels in IL-6-induced OSCC cells (Fig. 3G). Overall, our results uncovered that inhibition of JAK2/STAT3 pathway could restrain proliferation and NLRP3 inflammasome activation, which was mediated by IL-6 in OSCC cells.

### Silencing of Sox4 markedly suppressed proliferation and NLRP3 inflammasome activation in IL-6-treated OSCC cells

**Fig. 4:**
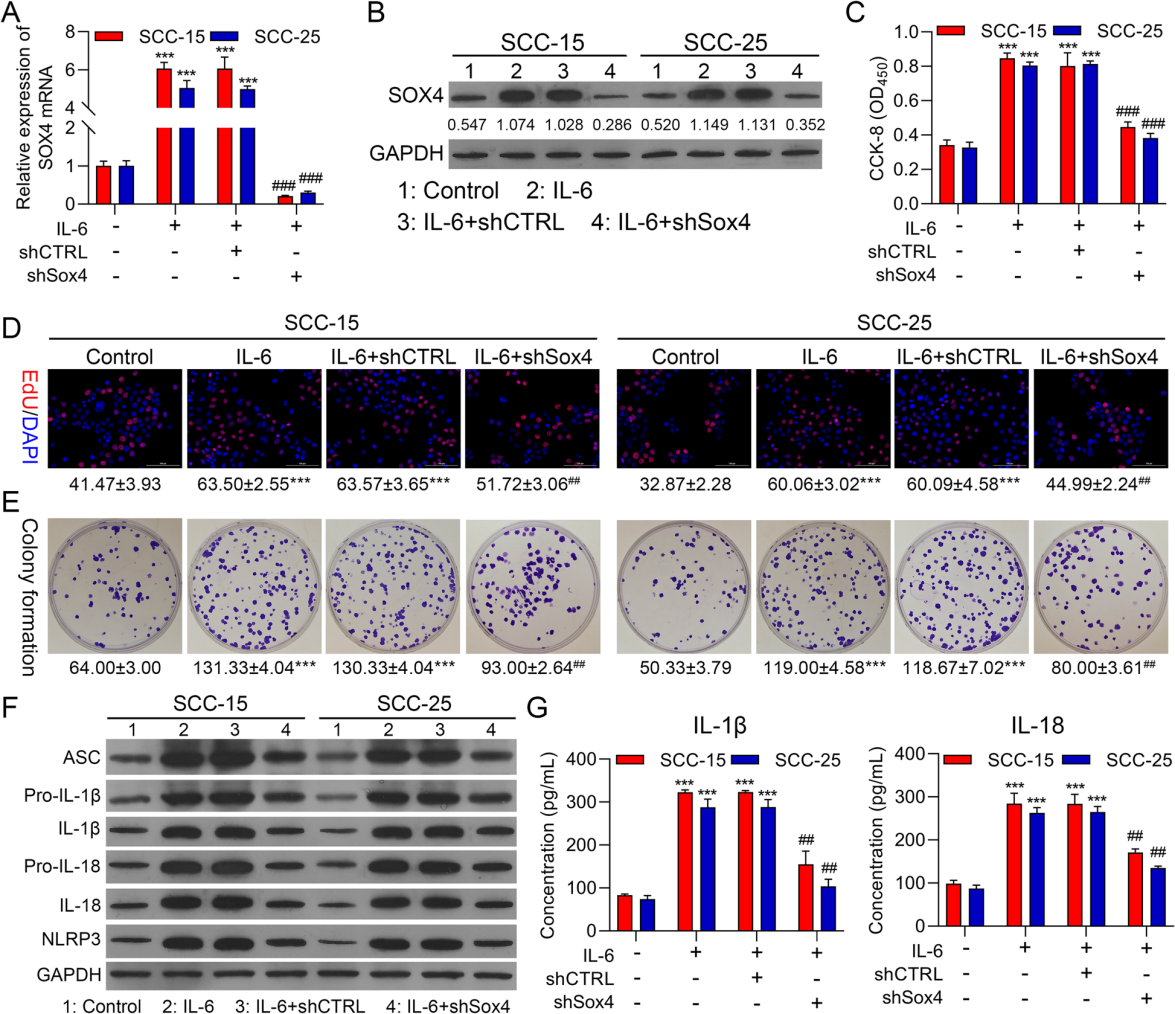
Silencing of Sox4 markedly suppressed proliferation and NLRP3 inflammasome activation in IL-6-treated OSCC cells. shCTRL and shSox4 were constructed and transfected into SCC-15 and SCC-25 cells, which were also disposed of 25 ng/ml IL-6. RT-qPCR (**A**) and Western blot (**B**) displayed the change in Sox4 expression. Cell proliferation was confirmed with (**C**) CCK-8, (**D**) EdU staining and (**E**) colony formation. (**F**) Western blot was adopted to verify the expression changes of inflammation-related proteins. (**G**) The changes of IL-1β and IL-18 levels were also evaluated with ELISA kits. *** P < 0.001 vs. IL-6 group; ## *P* < 0.01, ### *P* < 0.001 vs. IL-6 + shCTRL group. CTRL, control

On account of the induction of Sox4 expression by IL-6, we also verified whether Sox4 can participate in the promoting effect of IL-6 on proliferation and NLRP3 inflammasome activation in OSCC cells. In this part, Sox4 was silenced in IL-6-treated SCC-15 and SCC-25 cells. As denoted in Fig. 4A and 4B, Sox4 silencing could signally downregulate Sox4 in IL-6-treated OSCC cells. And the noteworthy increase of cell proliferation, which was induced by IL-6, also could be observably reversed by Sox4 silencing in OSCC cells (Fig. 4C-4E). Additionally, our results uncovered that and NLRP3-related proteins also could be memorably downregulated by Sox4 silencing in IL-6-treated OSCC cells (Fig. 4F). Simultaneously, ELISA results further displayed that Sox4 silencing could markedly lower IL-1β and IL-18 levels, which were increased by IL-6 in OSCC cells (Fig. 4G). In short, we verified that Sox4 was crucial in the induction of IL-6 to proliferation and NLRP3 inflammasome activation in OSCC cells.

### Upregulation of Sox4 prominently resisted blocking STAT3 mediated suppression of proliferation and NLRP3 inflammasome activation in IL-6-induced OSCC cells

**Fig. 5:**
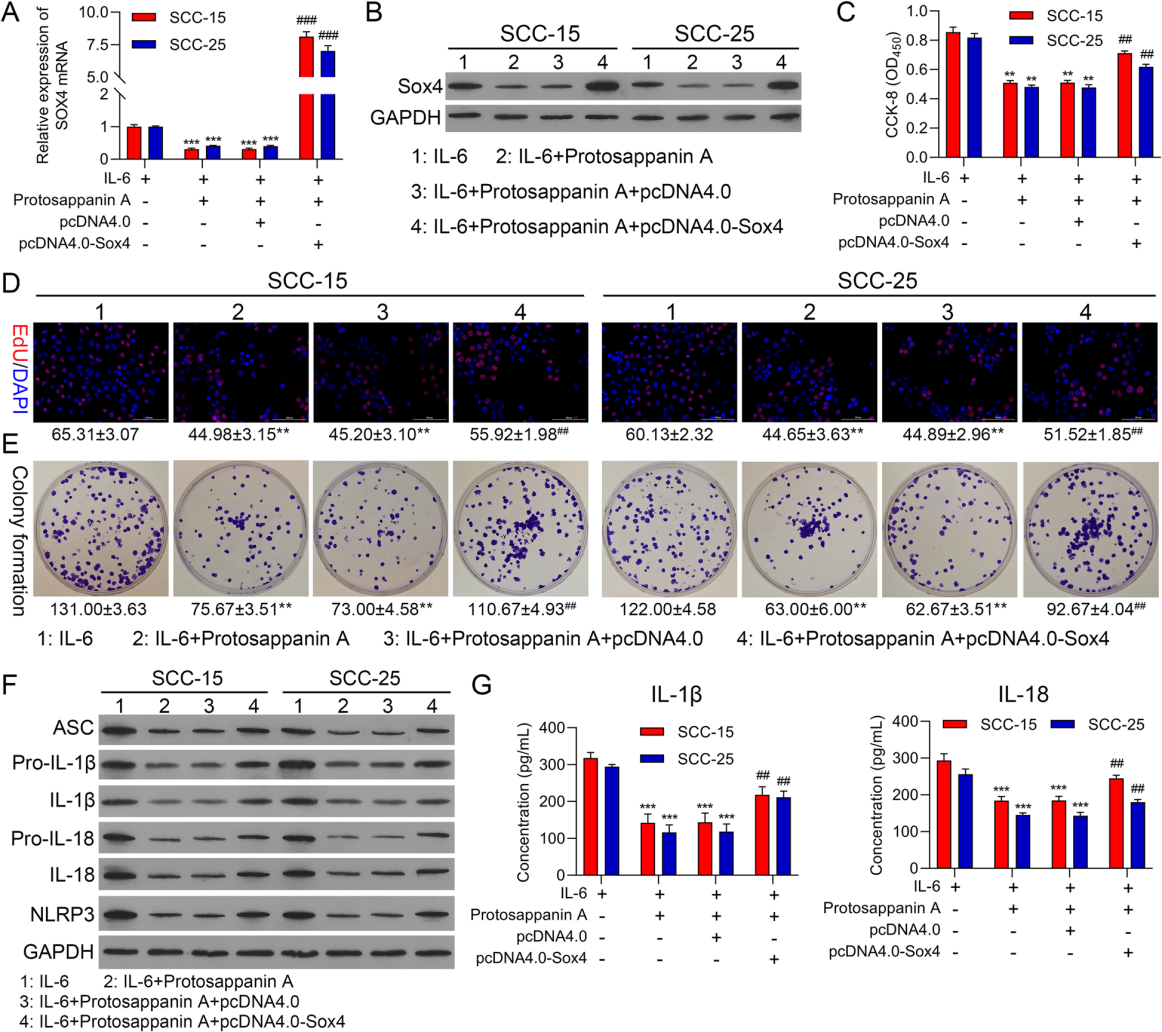
Upregulation of Sox4 prominently resisted blocking STAT3 mediated suppression of proliferation and NLRP3 inflammasome activation in IL-6-induced OSCC cells. SCC-15 and SCC-25 cells were dealt with 25 ng/ml IL-6 or 25 μmol/L Protosappanin A, and transfected with Sox4 overexpression plasmid. The expression change of Sox4 was confirmed with RT-qPCR (**A**) and Western blot (**B**). (**C**) CCK-8, (**D**) EdU staining and (**E**) colony formation was applied to exhibit the change in cell proliferation. (**F**) Western blotting analysis of the inflammation-related proteins. (**G**) ELISA analysis of IL-1β and IL-18 levels. ** P < 0.01, *** P < 0.001 vs. IL-6 group; ## *P* < 0.01, ### *P* < 0.001 vs. IL-6 + Protosappanin A group

As proved by the above results, both Sox4 and STAT3 pathway could be upregulated by IL-6 during OSCC progression. In this part, we further confirmed the regulatory relationship between Sox4 and STAT3 in OSCC progression. Sox4 was overexpressed in SCC-15 and SCC-25 cells, which also processed with Protosappanin A and IL-6. And our data represented that Protosappanin A could notably reduce Sox4 expression, while the downregulation of Sox4 mediated by Protosappanin A could also be repressed by Sox4 overexpression in IL-6-treated OSCC cells (Fig. 5A and 5B). Similarly, we found that cell proliferation was prominently reduced in IL-6 + Protosappanin A group compared to that in IL-6 group; and Sox4 overexpression also could dramatically accelerate cell proliferation mediated by Protosappanin A in IL-6-treated OSCC cells (Fig. 5C-5E). And western blot data signified that Protosappanin A observably decreased and NLRP3-related proteins, while the decrease in these proteins could be signally reversed by Sox4 overexpression in IL-6-treated OSCC cells (Fig. 5F). Likewise, ELISA results manifested that Sox4 overexpression also could attenuate the downregulation of IL-1β and IL-18 levels mediated by Protosappanin A in IL-6-treated OSCC cells (Fig. 5G). On balance, these findings proved that Sox4, as a downstream gene of STAT3 pathway, could participate in proliferation and NLRP3 inflammasome activation of IL-6-induced OSCC cells.

### STAT3 could signally regulate Sox4 expression in a targeted manner

**Fig. 6:**
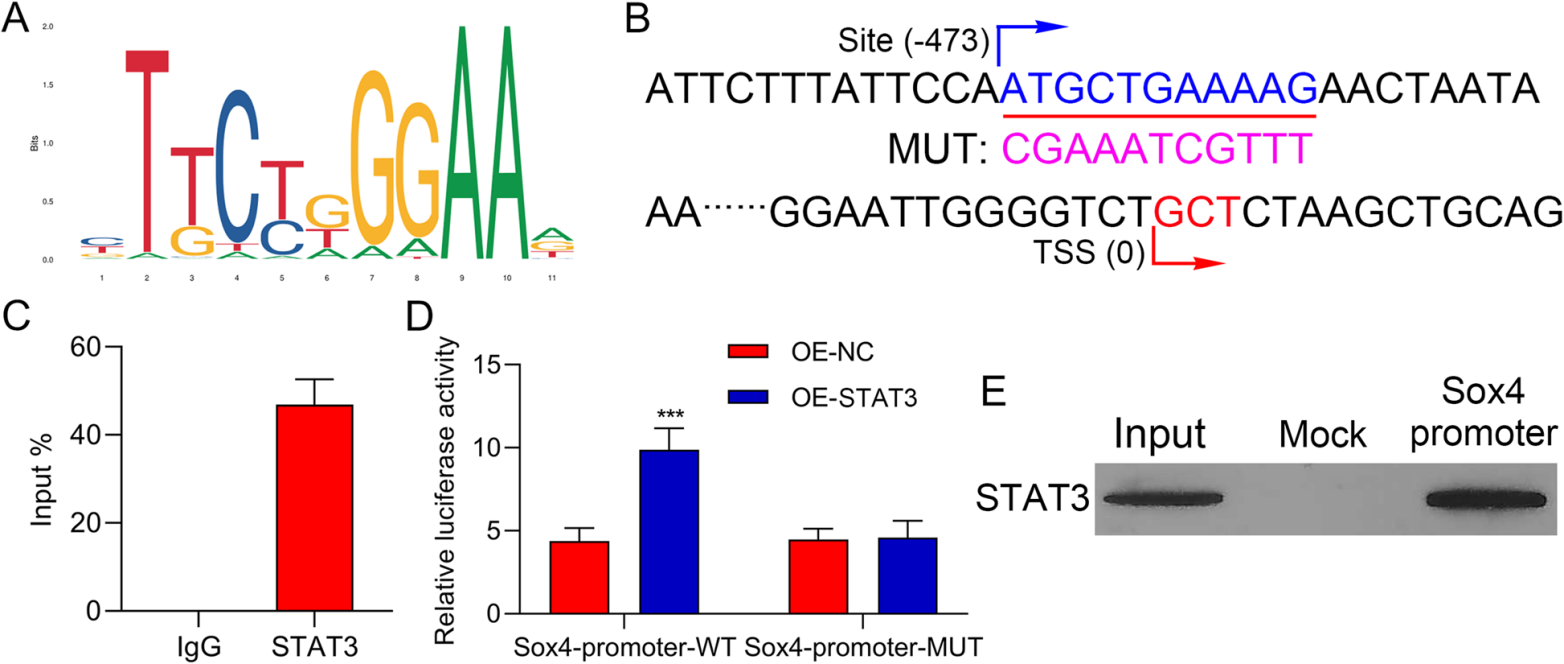
STAT3 could signally regulate Sox4 expression in a targeted manner. (**A**) Identification sites on STAT3 gene were exhibited. (**B**) Binding sites of STAT3 on Sox4 promoter and the sequence of mutations. (**C**) The regulatory relationship between STAT3 and Sox4 was certified with ChIP. (**D**) The targeted regulation of Sox4 by STAT3 was verified with Dual luciferase reporter gene assay. (**E**) DNA Pull-down was applied to test the regulatory correlation between STAT3 expression and Sox4 promoter. *** P < 0.001

Next, we further confirmed the targeted regulation of STAT3 to Sox4. Through analysis, we first determined identification sites on STAT3 gene (Fig. 6A). And based on the binding sites between STAT3 and Sox4 promoter, we also constructed the WT- and Mut- Sox4 promoter pGL3 plasmids (Fig. 6B). And ChIP data indicated that relative to IgG group, the expression intensity of Sox4 was memorably enhanced in STAT3 group, suggesting that Sox4 promoter could be combined with STAT3 protein (Fig. 6C). And STAT3 overexpression could notably increase the luciferase activity in WT-Sox4 promoter, while the luciferase activity was unaltered in Mut-Sox4 promoter (Fig. 6D). And DNA Pull-down data exhibited that the protein level of STAT3 was markedly enriched in Sox4 promoter group versus that in mock group (Fig. 6E). Generally, these results testified that STAT3 could target Sox4.

### Knockdown of NLRP3 notably weakened proliferation and NLRP3 inflammasome activation in IL-6-mediated OSCC cells

**Fig. 7:**
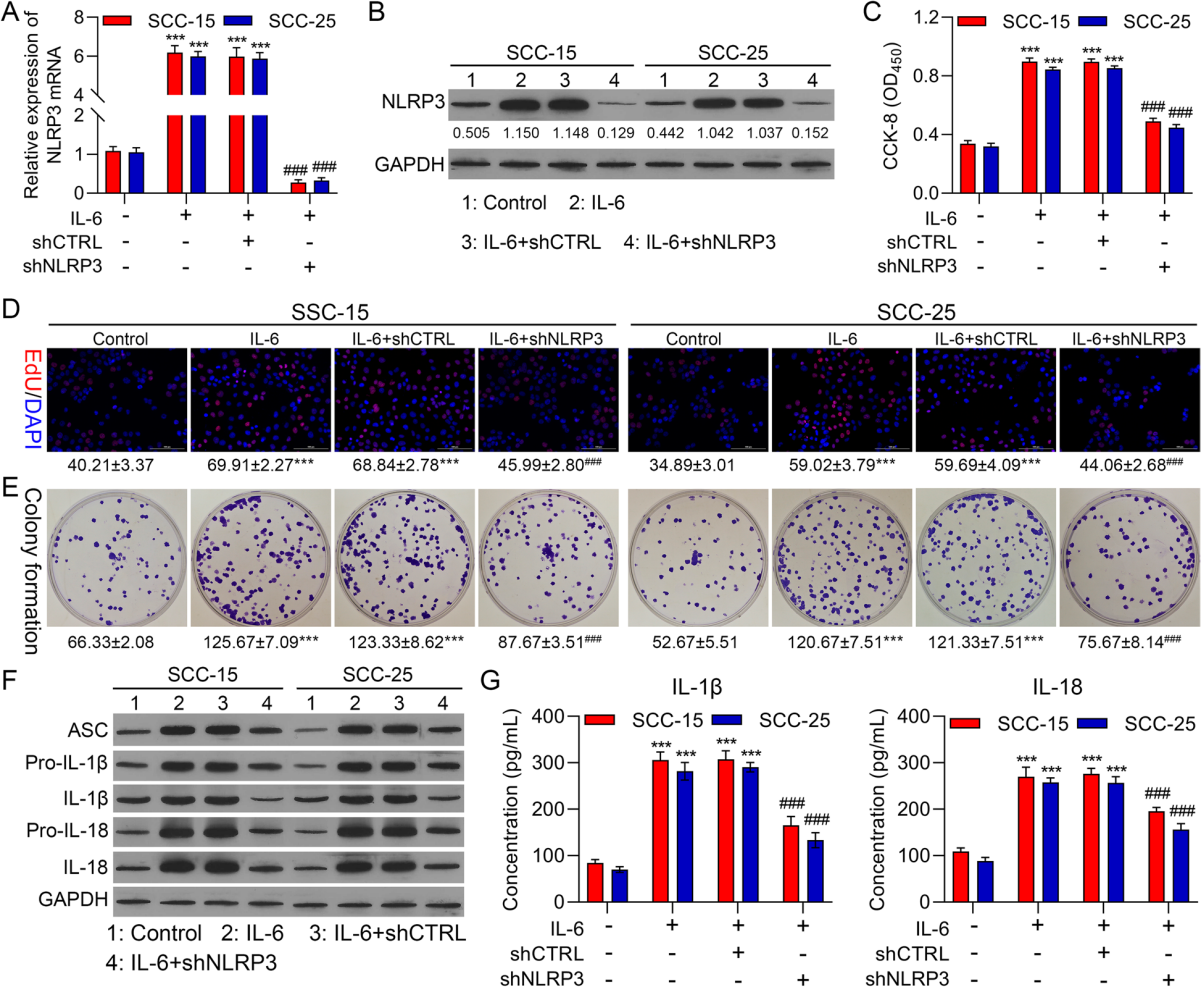
Knockdown of NLRP3 notably weakened proliferation and NLRP3 inflammasome activation in IL-6-mediated OSCC cells. NLRP3 was silenced in SCC-15 and SCC-25 cells, which were addressed with 25 ng/ml IL-6. NLRP3 expression was assessed through RT-qPCR (**A**) and western blot (**B**). Cell proliferation was determined via (**C**) CCK-8, (**D**) EdU staining and (**E**) colony formation. (**F**) The expressions of the inflammation-related proteins were monitored with Western blot. (**G**) ELISA kits were applied to identify IL-1β and IL-18 levels. *** P < 0.001 vs. IL-6 group; ### *P* < 0.001 vs. IL-6 + shCTRL group

Subsequently, we further investigated whether NLRP3 is a key regulatory protein in IL-6-mediated OSCC cell proliferation. We silenced NLRP3 in IL-6-treated SCC-15 and SCC-25 cells. And our data denoted that the expression of NLRP3 could be dramatically diminished by NLRP3 silencing in IL-6-treated OSCC cells (Fig. 7A and B). And cell proliferation also could be prominently depressed by NLRP3 silencing in IL-6-treated OSCC cells (Fig. 7C-E). Additionally, and NLRP3-related proteins mediated by IL-6 also could be markedly downregulated by NLRP3 silencing in IL-6-treated OSCC cells (Fig. 7F). Similarly, the data from ELISA also signified that silence of NLRP3 could signally lower the concentrations of IL-1β and IL-18 in IL-6-treated OSCC cells (Fig. 7G). Thus, the data disclosed that IL-6 might induce OSCC progression through NLRP3 pathway.

### Sox4 silencing dramatically repressed proliferation and NLRP3 inflammasome activation by downregulating NLRP3 in IL-6-induced OSCC cells

**Fig. 8:**
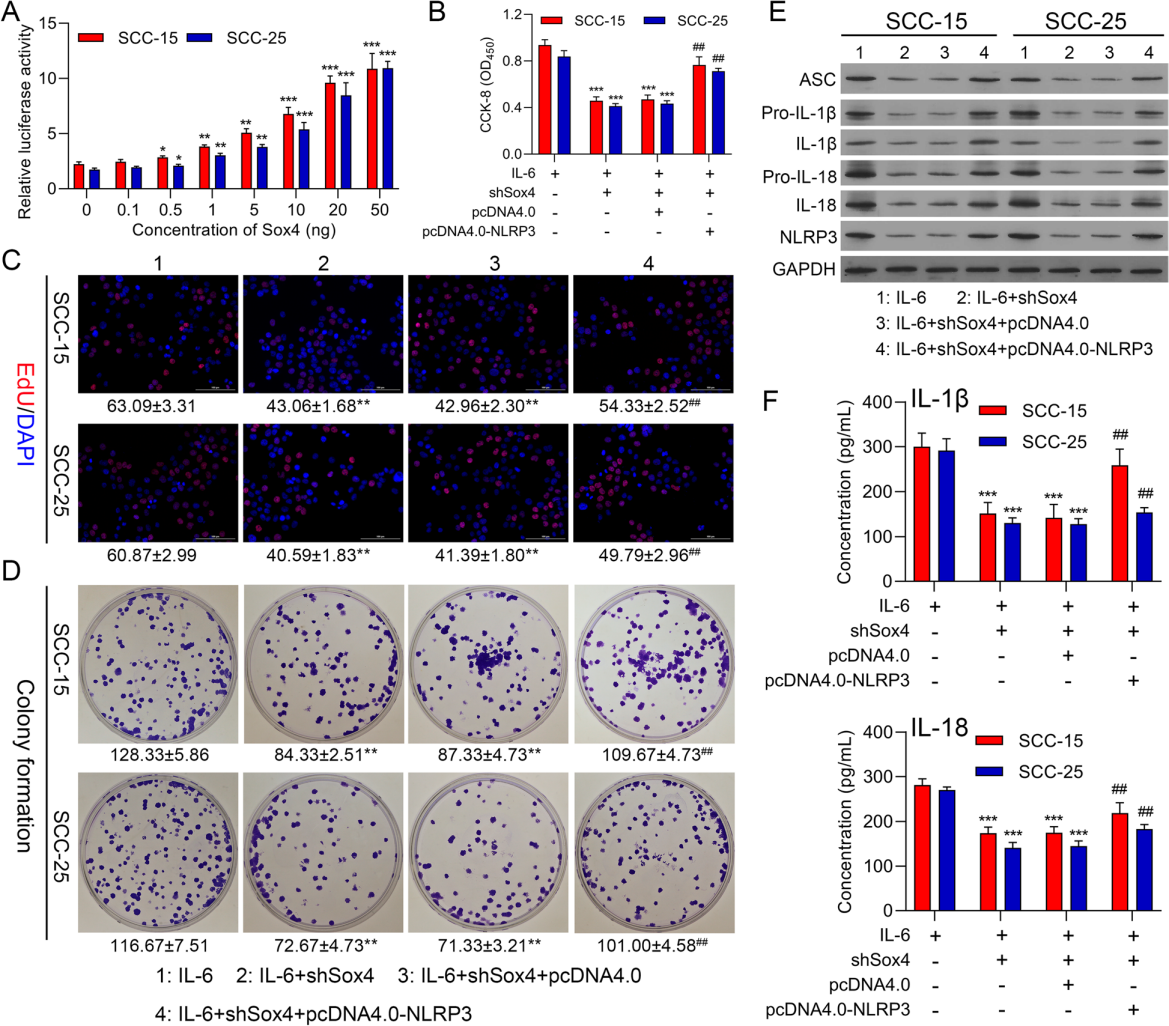
Sox4 silencing dramatically repressed proliferation and NLRP3 inflammasome activation by downregulating NLRP3 in IL-6-induced OSCC cells. (**A**) The influence of Sox4 on NLRP3 promoter activity was examined using Dual luciferase reporter gene assay, * P < 0.05, ** P < 0.01, *** P < 0.001. (**B**) CCK-8, (**C**) EdU staining, and (**D**) colony formation were conducted to test cell proliferation in IL-6-treated SCC-15 and SCC-25 cells, which were transfected with shSox4 or/and NLRP3 overexpression plasmid, respectively. (**E**) Western blot was utilized to investigate the impacts of Sox4 silencing and NLRP3 overexpression on the levels of the inflammation-related proteins in IL-6-induced OSCC cells. (**F**) IL-1β and IL-18 levels were analyzed using ELISA kits in the processed OSCC cells. *** P < 0.001 vs. IL-6 group; ## *P* < 0.01 vs. IL-6 + shSox4 group

Furthermore, we also verified the regulatory relationship between Sox4 and NLRP3 in OSCC progression. As represented in Fig. 8A, as the concentration of Sox4 increased, the luciferase activity of NLRP3 also observably increased in OSCC cells. Besides, our data manifested that Sox4 silencing could memorably suppress cell proliferation, while this suppression of cell proliferation mediated by Sox4 silencing also could be reversed by NLRP3 overexpression in IL-6-treated OSCC cells (Fig. 8B-D). Next, Western blot data displayed that Sox4 silencing also could dramatically downregulate ASC, pro-IL-1β, IL-1β, pro-IL-18, IL-18, and NLRP3 in IL-6-treated OSCC cells, which also could be weakened by NLRP3 overexpression (Fig. 8E). ELISA results also exhibited that Sox4 silencing could notably decrease IL-1β and IL-18 levels through NLRP3 in IL-6-induced OSCC cells (Fig. 8F). Consequently, we suggested that silence of Sox4 could prevent OSCC progression via NLRP3.

### IL-6 notably accelerated tumor growth and activated JAK2/STAT3/Sox4/NLRP3 pathway in nude mouse xenografts of OSCC

**Fig. 9:**
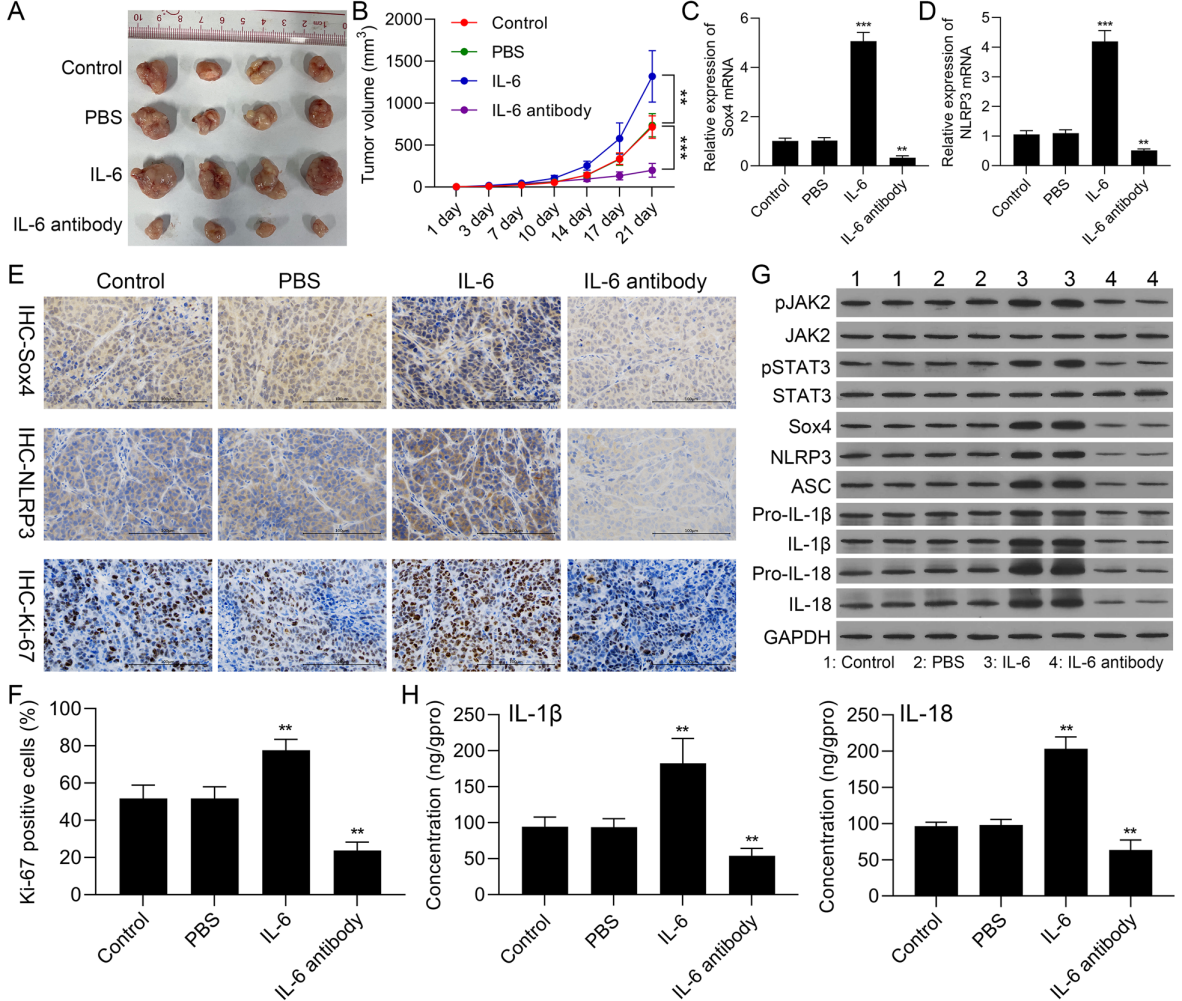
IL-6 notably accelerated tumor growth and activated JAK2/STAT3-Sox4-NLRP3 pathway in nude mouse xenografts of OSCC. SCC-15 cells were applied to construct nude mouse xenografts, which were intervened with IL-6 or anti-IL-6. (**A**) Subcutaneous tumors were dissected and photographed at the end of day 21. (**B**) Tumor growth curve was presented after xenotransplantation. Sox4 (**C**) and NLRP3 (**D**) expressions were certified with RT-qPCR. (**E**) Immunohistochemical staining of Sox4, NLRP3, and Ki-67 expressions in the tumors. (**F**) Ki-67 positive cells were counted. (**G**) Western blotting analysis of JAK2, STAT3, Sox4, and NLRP3 inflammasome activation-related proteins. (**H**) IL-1β and IL-18 levels were confirmed using ELISA kits in the serum of nude mice. ** P < 0.01, *** P < 0.001

In line with the action and mechanism of IL-6 in vitro OSCC cells, we further determined these effects of IL-6 in the OSCC xenograft tumor. Nude mice xenografts were first constructed with SCC-15 cells through subcutaneous injection, and then mice were intervened with IL-6 or anti-IL-6. As indicated in Fig. 9A and B, IL-6 treatment could observably increase the growth of the tumors, and the processing of anti-IL-6 could signally reduce the growth of the tumors in mice. Besides, we revealed that IL-6 could memorably upregulate Sox4 and NLRP3, and anti-IL-6 could markedly downregulate Sox4 and NLRP3 in the tumors of mice (Fig. 9C-E). And Ki-67 positive cells also could be increased by IL-6 and decreased by anti-IL-6 in the tumors of mice (Fig. 9E and F). Moreover, Western blotting results signified that IL-6 dramatically elevated pJAK2, pSTAT3, Sox4, NLRP3, ASC, pro-IL-1β, IL-1β, pro-IL-18, and IL-18 expressions, and anti-IL-6 observably had the opposite effect to IL-6 on the expressions of these proteins in the tumors of mice (Fig. 9G). And ELISA results disclosed that relative to the serum of mice in PBS group, IL-1β and IL-18 levels were notably elevated in IL-6 group and prominently lowered in anti-IL-6 group (Fig. 9H). In summary, we demonstrated that IL-6 also could enhance the growth in nude mouse xenografts of OSCC, which might be relevant to JAK2/STAT3/Sox4/NLRP3 pathway.

### Sox4 knockdown obviously weakened the induction of IL-6 on the tumor growth and inflammation in nude mouse xenografts of OSCC

**Fig. 10:**
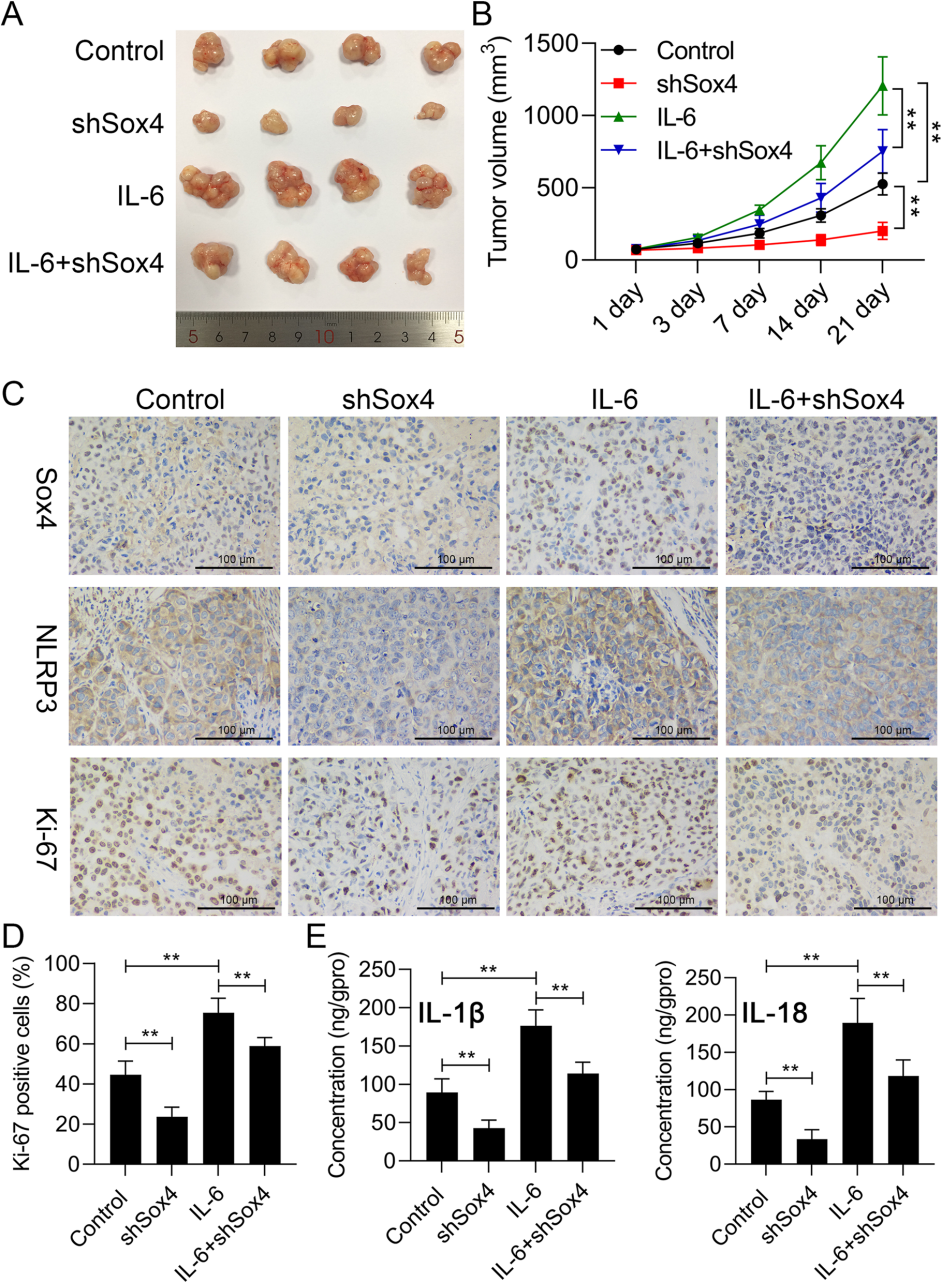
Sox4 knockdown obviously weakened the induction of IL-6 on the tumor growth and inflammation in nude mouse xenografts of OSCC. SCC-15 cells processed with shSox4 or/and IL-6 were subcutaneously injected into nude mice. (**A**) After 3 weeks, groups of tumors were presented. (**B**) The volume of the tumor was counted in each group. (**C**) Immunohistochemical staining of Sox4, NLRP3, and Ki-67 expressions in each group. (**D**) Ki-67 positive cells were calculated. (**E**) ELISA kits were applied to analyze IL-1β and IL-18 levels in the serum of nude mice

Furthermore, we also explore whether the inhibition of Sox4 can affect the tumor-bearing mice mediated by IL-6 in vivo. As exhibited in Fig. 10A and Fig. 10B, in contrast to IL-6, Sox4 knockdown could result in a remarkable reduction in tumor volume, meanwhile, Sox4 knockdown also could reduce the increase of tumor volume mediated by IL-6. Next, IHC results signified that Sox4 knockdown could signally downregulate Sox4 and NLRP3, and reduce Ki-67 positive cells in the tumors of mice, which also could be memorably reversed by IL-6, and the elevation of Sox4 and NLRP3 expressions and Ki-67 positive cells caused by IL-6 also could be prominently attenuated by Sox4 knockdown (Fig. 10C and Fig. 10D). Moreover, ELISA data revealed that Sox4 knockdown could dramatically reduce IL-1β and IL-18 levels in the tumors of mice, which also could be markedly weakened by IL-6, and the increase of IL-1β and IL-18 levels mediated by IL-6 also could be memorably reduced by Sox4 knockdown (Fig. 10E). Therefore, we proved that inhibition of Sox4 also can obviously prevent the growth and inflammation of tumor bearing mice mediated by IL-6.

## Discussion

The incidence of head and neck cancer is relatively high, ranking 6th in the incidence of systemic malignant tumors, among which 90% are OSCC, and the prognosis is poor [2, 25]. However, the pathogenesis of OSCC is not clear at present. Tumor-associated inflammation is known as the 7th biological feature of malignant tumors [26]. On the one hand, chronic inflammation has key roles in tumor genesis, development, migration, and invasion through NF-κB-IL-6-STAT pathway; on the other hand, chronic inflammation can recruit immune cells and inflammatory cells to the tumor tissue, and with the development of tumor, the function of these cells evolved from inhibition and immune surveillance to promoting tumor cell proliferation [27]. IL-6, as a multifunctional molecule, is involved in immune and inflammatory responses [7]. Besides, IL-6 has been reported to be overexpressed in patients with cancers, including OSCC, which is associated with poor prognosis [11, 28]. IL-6 also could induce tumor cell growth, metastasis, and angiogenesis through IL-6R-mediated pathways [7]. In our study, we also testified that IL-6 was upregulated in OSCC tissues, and connected with inflammatory cytokines (IL-1β and IL-18). Besides, IL-6 also could upregulate IL-6R and accelerate proliferation of OSCC cells, which was consistent with previous research [29]. Moreover, we also certified that IL-6 could activate JAK2 and STAT3 pathways in OSCC cells.

It is reported that JAK2/STAT3 can receive extracellular stimulation signals through inflammatory cytokine receptors, then induce JAK2 and STAT3 phosphorylation and regulate the expression of downstream genes [30, 31]. JAK2/STAT3 has also been confirmed to be critically involved in the growth and metastasis of multiple cancers, including OCSS [32–34]. In our study, we proved that JAK2 inhibitor (Fedratinib) and STAT3 inhibitor (Protosappanin A) could notably attenuate the induction of IL-6 on the proliferation and inflammation of OCSS cells. thus, we demonstrated that IL-6 could accelerate OSCC progression by JAK2 and STAT3 pathway.

To further investigate the possible downstream genes regulated by STAT3, we reviewed vast literature. It was reported that STAT3 can induce abnormal proliferation of tumor cells through regulation of BCL-2, FAS, survivin and CyclinD1 [35, 36]. STAT3 can accelerate neovascularization by regulating VEGF and bFGF [37]. Knockdown of STAT3 can increase NF-κB promoter activity and enhance NF-κB accumulation in the nucleus [38]. STAT3 silencing also could reduce the expressions of cytokines IL-6, IL-1β and inflammatory mediators ICAM1 and COX2 in the tumor microenvironment [39]. And MMP-2 and S100A4 may be the key targets of STAT3 involved in cancer metastasis [40, 41]. Besides, recent studies have also confirmed that STAT3 can participate in the cancer process by mediating the expression of Sox4 [42, 43]. Moreover, our previous research also revealed that Sox4 is significantly upregulated in oral lichen planus (OLP), OSCC versus OLP tissues, and Sox4 might be actively involved in the progression of OLP to OSCC, suggesting that Sox4 could induce the progression in OLP-associated OSCC [44]. Therefore, we selected Sox4 as a downstream gene of STAT3 for the next step in OSCC. SOX family is a novel family of genes that can regulate development [45]. SOX is characterized by a conserved HMG box, which can specifically bind to DNA sequences and is a key transcription regulatory factor [46]. And Sox4 is a transcription factor associated with development and differentiation [47]. Sox4 could induce tumorigenesis by endowing cancer cells with survivability, mobility, and invasiveness [48]. Several researches verified that Sox4 is a critical oncogene that is highly expressed in cancers, including prostate cancer [49], colorectal cancer [50], bladder cancer [51], and breast cancer [52], etc. Sox4 has also been testified to be associated with differentiation, metastasis, and chemoradioresistance in OSCC [53, 54]. Sox4 might have the potential to be a reliable prognostic factor for OSCC. While the regulatory pathways of Sox4 in OSCC progression have not been clearly elucidated. In our study, we also discovered that Sox4 was also upregulated and positively correlated with IL-6 in OSCC tissues. While it is unclear for the regulatory relationship between IL-6 and Sox4 in OSCC progression. Next, we further certified that Sox4 silencing could prevent proliferation and NLRP3 inflammasome activation mediated by IL-6 in OSCC cells. Moreover, we discovered that STAT3 could target Sox4, and STAT3 silencing could prevented OSCC progression by downregulating Sox4 in IL-6-induced OSCC cells. therefore, we demonstrated the IL-6/JAK2/STAT3/Sox4 pathway in OSCC.

Oral cavity, as a microenvironment colonized by more than 700 kinds of microorganisms, has been in the inflammatory environment with pathogenic bacteria for a long time [55]. About 25% of malignancies are reported to be associated with chronic inflammation or infection [56]. Chronic inflammation can cause the disappearance of cell growth inhibition, autonomic angiogenesis, apoptosis avoidance, transformation from benign to malignant, and enhancement of metastases [57]. In the early stage of tumor formation, reactive oxygen species (ROS) and active nitrogen substances produced by immune cell infiltration can lead to epigenetic changes of oncogenes and tumor suppressor genes, thus promoting tumor genesis [58]. During tumor metastasis, cytokines secreted by immune cells will enhance cell viability and invasion, resulting in transformation from epithelial cells to mesenchymal cells [59]. Therefore, the molecular mechanism between tumor and inflammation are key for the prevention and therapy of cancer. Inflammasome is a key molecular structure for the body to resist pathogens and recognize its own danger signal. NLRP3 inflammasome, as the core protein of NLRs family, is a member of innate immune system [60]. And its abnormal activation is associated with various chronic inflammation, mitochondrial diseases, and tumors [61]. The NLRP3 inflammasome consists of NLRP3, ASC, and pro-caspase-1. NLRP3 inflammasome can activate pro-caspase to form active caspase-1, which causes the maturation and secretion of IL-1β and IL-18, leading to inflammation [62]. IL-1β, as a class of pleiotropic pro-inflammatory cytokines, plays a key role in tumor development [63]. IL-1β can promote tumor growth and metastasis by inducing the expression of various metastasis-related factors, such as matrix metalloproteinases (MMPs), vascular endothelial growth factor (VEGF), inflammatory chemokines and growth factor genes [64]. IL-18 has been reported to exert antitumor effects through multiple pathways. IL-18 can induce proliferation and enhance the activity of T lymphocytes and NK cells [65]. IL-18 also promotes the production and secretion of cytokines such as IFN-γ, IL-2, GM-CSF, and TNF-α. And these cytokines are able to exert anti-tumor effects either directly by killing or by modulating immunity [64]. The relationship between NLRP3 inflammasome and malignant tumors is complex and has some tissue or cell specificity. Related studies also testified that NLRP3 inflammasome was increased in OSCC, and its expression was relevant to tumor stage and lymph node metastasis [66, 67]. And the activation of NLRP3 also could enhance the proliferation, migration, and invasion of OSCC cells [66]. However, the regulatory relationship between NLRP3 and Sox4 remains unclear. In our study, we also proved that IL-6 could induce OSCC progression through activation of NLRP3 and its downstream cascade reaction (secretion of IL-1β/IL-18). And silencing of NLRP3 also could prevent proliferation and NLRP3 inflammasome activation in IL-6-mediated OSCC cells. In addition, NLRP3 pathway could participate in IL-6-mediated OSCC process as a downstream pathway of Sox4.

## Conclusions

**Fig. 11:**
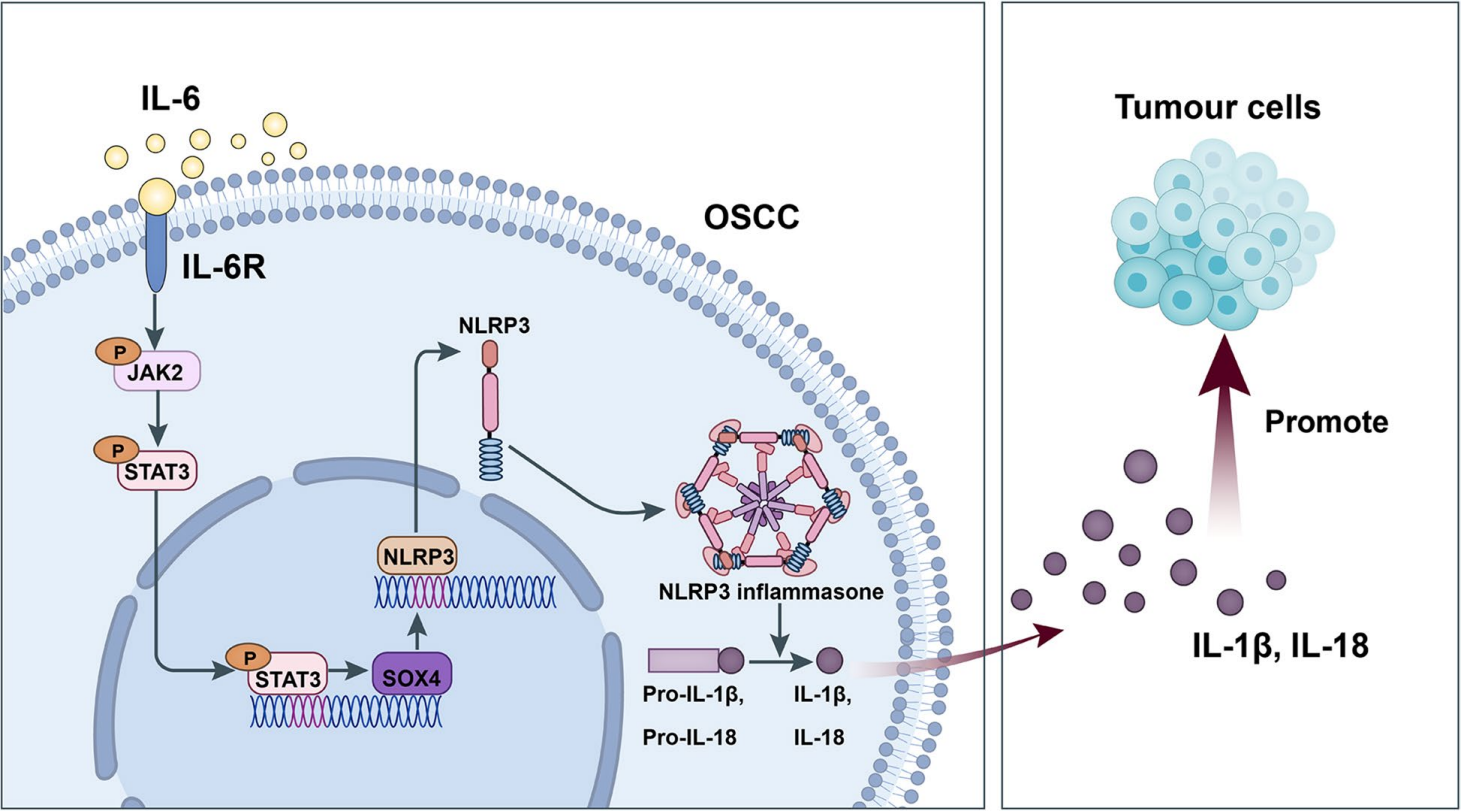
Schematic overview of the mechanistic basis for the observed study results. IL-6 plays an oncogenic role in OSCC progression by activating JAK2/STAT3/Sox4/NLRP3 pathway. IL-6 could accelerate OSCC cell proliferation by activating inflammasome via JAK2/STAT3/Sox4/NLRP3 pathways. STAT3 played as a transcription factor which positively regulated Sox4, and IL-6 promotes Sox4 expression by activating JAK2/STAT3 pathway. IL-6 could promote proliferation and NLRP3 inflammasome activation via JAK2/STAT3/Sox4 pathway in OSCC cells

This study demonstrated that IL-6, as an oncogene, is critical in the biological processes of OSCC. Besides, we verified a novel and effective molecular axis IL-6/JAK2/STAT3/Sox4/NLRP3 in OSCC progression (Fig. 11). Therefore, IL-6-mediated JAK2/STAT3/Sox4/NLRP3 might be the possible targets for OSCC therapy. However, the shortcoming of this study is that we did not analyze the main source of IL-6 in the OSCC microenvironment, and we will conduct this shortcoming in our future studies.

## Supplementary Information

**Additional file 1. **Supplementary Figure 1. IL-1ß and IL-18 were positively correlated with IL-6 concentration in OSCC patients. (**A**) The concentration of IL-1ß was determined with ELISA kits in the serum of normal, OLP, and OSCC patients. (**B**) Correlation analysis between IL-1ß concentration and IL-6 concentration in OSCC patients (*r* = 0.7054). (**C**) ELISA kit was utilized to test the concentration of IL-18 in the serum of normal, OLP, and OSCC patients. (**D**) Correlation analysis between IL-18 concentration and IL-6 concentration in OSCC patients (*r* = 0.6755). **** *P* < 0.0001. 

**Additional file 2. **Supplementary Figure 2. IL-6 observably increased IL-1ß and IL-18 levels in OSCC cells. The concentrations of IL-1ß and IL-18 were monitored using ELISA kits in SCC-15 and SCC-25 cells, which were administrated with 0, 0.1, 1, 5, 10, 25, 50 ng/mL IL-6. * *P* < 0.05, ** *P* < 0.01, *** *P* < 0.001. 

## Data Availability

The datasets used and analyzed during the current study are available from the corresponding author on reasonable request.

## Abbreviations

IL-6: Interleukin-6
OSCC: Oral Squamous Cell Carcinoma
RT-qPCR: Real-Time Quantitative PCR
ELISA: Enzyme Linked Immunosorbent Assay
JAK: Janus protein tyrosine kinase
OLP: Oral Lichen Planus
cDNA: Complementary DNA
ChIP: Chromatin immunoprecipitation
CCK-8: Cell Counting Kit-8
IHC: Immunohistochemistry
