# Supplementary figures and images for: Interleukin-6 mediated inflammasome activation promotes oral squamous cell carcinoma progression via JAK2/STAT3/Sox4/NLRP3 signaling pathway
Source: J Exp Clin Cancer Res. 2022 May 5;41:166. doi: 10.1186/s13046-022-02376-4 (PMC9069786; doi:10.1186/s13046-022-02376-4)

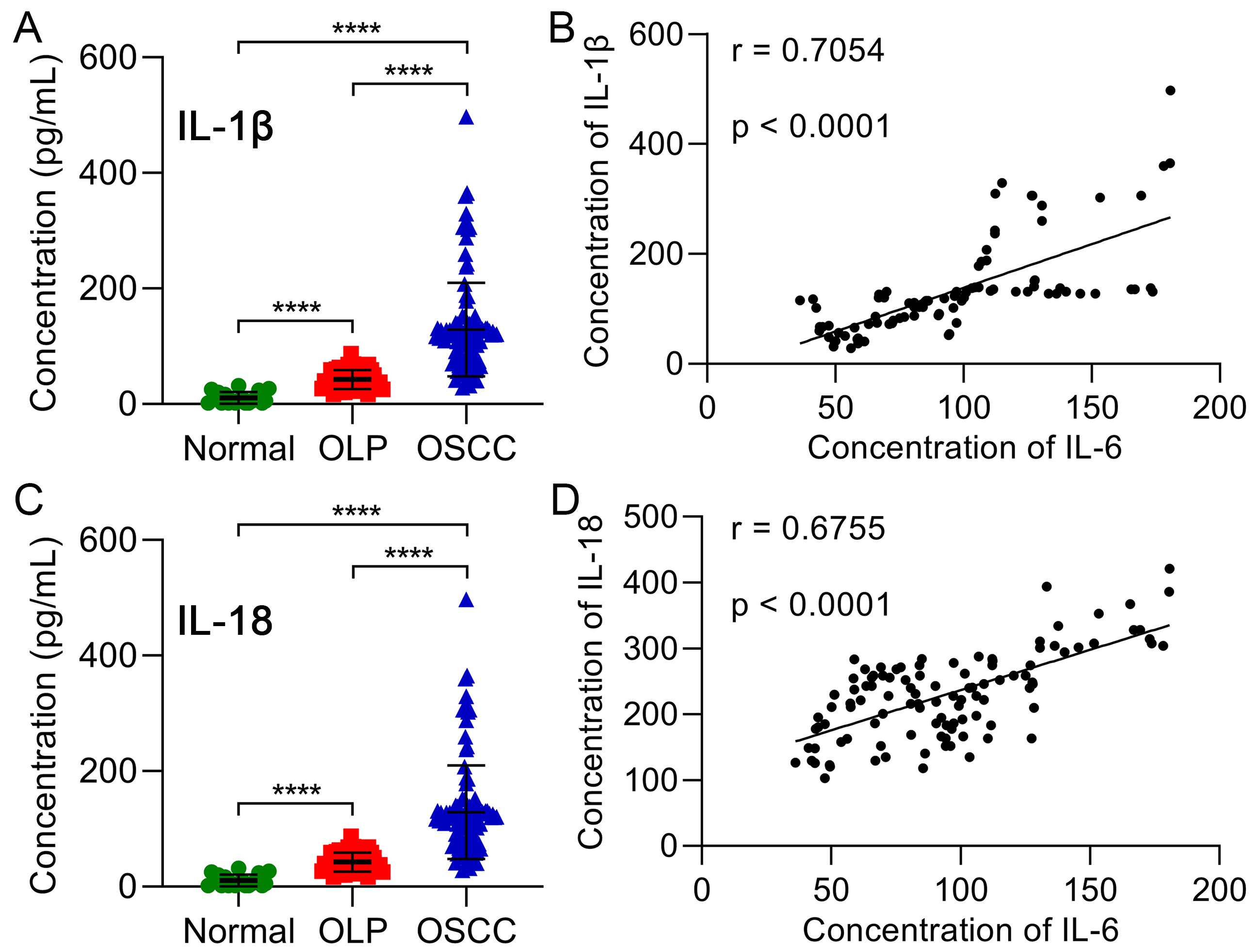

Supplement: Supplementary file 1 — Additional file 1. Supplementary Figure 1. IL-1ß and IL-18 were positively correlated with IL-6 concentration in OSCC patients. (A) The concentration of IL-1ß was determined with ELISA kits in the serum of normal, OLP, and OSCC patients. (B) Correlation analysis between IL-1ß concentration and IL-6 concentration in OSCC patients (r = 0.7054). (C) ELISA kit was utilized to test the concentration of IL-18 in the serum of normal, OLP, and OSCC patients. (D) Correlation analysis between IL-18 concentration and IL-6 concentration in OSCC patients (r = 0.6755). **** P < 0.0001. [file 13046_2022_2376_MOESM1_ESM.tif]

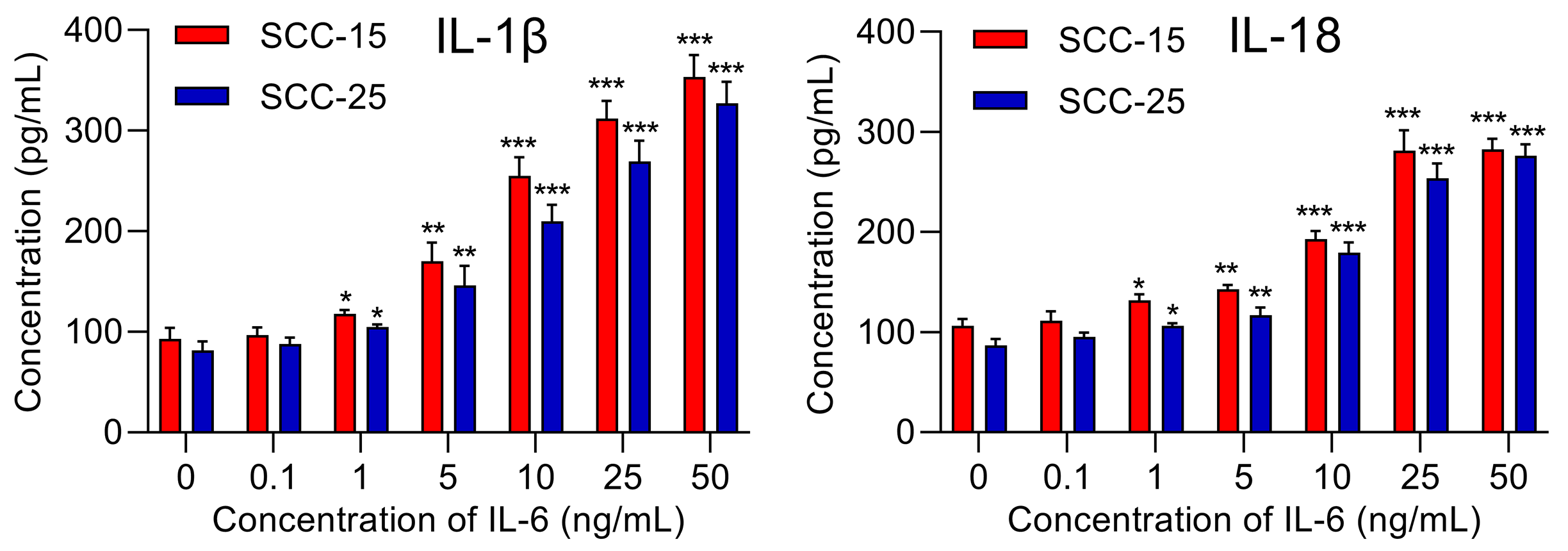

Supplement: Supplementary file 2 — Additional file 2. Supplementary Figure 2. IL-6 observably increased IL-1ß and IL-18 levels in OSCC cells. The concentrations of IL-1ß and IL-18 were monitored using ELISA kits in SCC-15 and SCC-25 cells, which were administrated with 0, 0.1, 1, 5, 10, 25, 50 ng/mL IL-6. * P < 0.05, ** P < 0.01, *** P < 0.001. [file 13046_2022_2376_MOESM2_ESM.tif]
